# Impaired glymphatic drainage underlying obstructive sleep apnea is associated with cognitive dysfunction

**DOI:** 10.1007/s00415-022-11530-z

**Published:** 2023-01-20

**Authors:** Jiuqi Wang, Yiming Tian, Chi Qin, Lin Meng, Renyi Feng, Shuqin Xu, Yanping Zhai, Dongxiao Liang, Rui Zhang, Haiyan Tian, Han Liu, Yongkang Chen, Yu Fu, Pei Chen, Qingyong Zhu, Junfang Teng, Xuejing Wang

**Affiliations:** 1 Department of Neurology, The First Affiliated Hospital of Zhengzhou University, Zhengzhou, 450052 Henan China; 2 Institute of Parkinson and Movement Disorder, Zhengzhou University, Zhengzhou, 450052 Henan China

**Keywords:** Obstructive sleep apnea, Glymphatic, Cognitive impairment, Dynamic contrast-enhanced magnetic resonance imaging

## Abstract

Obstructive sleep apnea (OSA) is highly prevalent but easily undiagnosed and is an independent risk factor for cognitive impairment. However, it remains unclear how OSA is linked to cognitive impairment. In the present study, we found the correlation between morphological changes of perivascular spaces (PVSs) and cognitive impairment in OSA patients. Moreover, we developed a novel set of dynamic contrast-enhanced magnetic resonance imaging (DCE-MRI) methods to evaluate the fluid dynamics of glymphatic drainage system. We found that the inflow and outflow parameters of the glymphatic drainage system in patients with OSA were obviously changed, indicating impairment of glymphatic drainage due to excessive perfusion accompanied with deficient drainage in OSA patients. Moreover, parameters of the outflow were associated with the degree of cognitive impairment, as well as the hypoxia level. In addition, continuous positive airway pressure (CPAP) enhances performance of the glymphatic drainage system after 1 month treatment in OSA patients. We proposed that ventilation improvement might be a new strategy to ameliorate the impaired drainage of glymphatic drainage system due to OSA-induced chronic intermittent hypoxia, and consequently improved the cognitive decline.

**Supplementary Information:**

The online version contains supplementary material available at 10.1007/s00415-022-11530-z.

## Introduction

The glymphatic drainage system is a highly organized fluid clearance pathway between cerebrospinal fluid (CSF) and interstitial fluid (ISF), which subserves the CSF influx into the brain through periarterial spaces (PASs), and ISF efflux from the brain into perivenous spaces (PVESs) that eventually reaches the subarachnoid space [1–3], which potentially drains the metabolic wastes from the brain [4]. Studies show that the dysfunction of glymphatic drainage system causes cognitive impairment[5], and it has been clarity that the dysfunction of glymphatic drainage was associated with the impaired cognitive function in Alzheimer's disease (AD) and Parkinson’s disease (PD) [6, 7].

Obstructive sleep apnea (OSA) is a common sleep related disorder with the symptoms of upper airway collapse, apnea, hypoxia and recurrent arousals, accompanied by decline in cognitive function [8, 9]. Sleep-disordered breathing was linked to an increased risk of cognitive impairment and AD in the elderly population [10–14]. However, the mechanism and prognosis of cognitive impairment in OSA patients remain unclear. Treatment with continuous positive airway pressure (CPAP) is the main treatment for OSA, which was confirmed to delay progression and delay the decline of cognitive function [15–18]. The mechanism of improvement in cognitive impairment, however, is unknown.

In the present work, we used the MRI axial T2 sequence to compare morphological changes of glymphatic drainage system in patients with OSA and normal controls (NCs). The diagnostic accuracy of morphological changes was analyzed and the correlations between the glymphatic drainage system morphological changes and cognitive impairment were confirmed. In addition, we developed a set of novel DCE-MRI techniques for assessing the fluid dynamics in the glymphatic drainage system. We investigated that the dysfunction of outflow drainage of the glymphatic drainage system was correlated with the cognitive decline. Moreover, treatment with CPAP could promote the outflow drainage of the glymphatic dynamic and further improve the cognitive function in OSA patients.

## Materials and methods

### Approval and patient informed consent

This study was authorized by the Institutional Ethics Committees of The First Affiliated Hospital of Zhengzhou University (2022-KY-0282-004). Informed and signed consent was obtained from all participants or their legal guardians.

### Participants

52 OSA patients were recruited based on the following criteria: (1) have no other medical illnesses, such as a history of high blood pressure, cardiopulmonary disease, neurological, kidney, or liver diseases, diabetes, or cancer, as well as no prior upper airway surgery, pulmonary surgery, or snoring therapy, (2) have no structural abnormalities on brain MRI with visual inspections, and (3) diagnosed with OSA on apnea–hypopnea index (AHI) ≥ 5 measured by polysomnography (PSG) monitoring combined with symptoms, such as sleepiness or chronic snoring. In addition, 56 healthy participants were also recruited through the Physical Examination Center of the First Affiliated Hospital of Zhengzhou University. Physical examination, ECG, laboratory testing, such as blood and urine routine, liver and kidney function, MRI, and magnetic resonance angiography scans, all revealed that they were in good conditions. All healthy participants had with no history of medical or neurological disorders, as determined by two attending neurologists and a psychiatrist.

All the participants underwent a clinical assessment and were investigated by two board-certified neurologists who had experience with neurodegenerative diseases. We applied the Mini-mental State Examination (MMSE) and the Montreal Cognitive Assessment (MoCA) to assess cognitive function, the Pittsburgh Sleep Quality Index (PSQI) and the Epworth sleepiness scale (ESS) for the sleep assessment.

### PSG

PSG was used to capture the electroencephalogram, bilateral electro-oculograms, surface EMG, electrocardiogram, chest and abdominal wall movements, and oxygen saturation (SaO_2_) using a pulse oximeter in OSA patients.

According to predetermined standards, respiratory episodes were scored. [19, 20]. Apnea was defined as a complete halt of airflow lasting at least 10 s. Hypopneas were defined as a noticeable decrease in airflow lasting at least 10 s and accompanied by at least 3% desaturation. The total number of obstructive apneas and obstructive hypopneas per hour of sleep was used to defined the apnea–hypopnea index (AHI). Total oxygen desaturation index (ODI) was outlined as the sum of all desaturations of at least 3% for each hour of total sleep. According to frequently used clinical cutoffs, the following groups were established: no OSA (AHI < 5); mild-moderate OSA group (AHI ≥ 5 but < 30); and severe OSA group (AHI ≥ 30). Moreover, the hypoxemia severity groups were established: mild hypoxemia group (lowest SaO_2_ (LSaO_2_) ≥ 80% but < 90); and severe hypoxemia group (LSaO_2_ < 80%)[21].

### Transcranial doppler

For the TCD examination, a transcranial doppler (TCD) analyzer (EMS-9A, Shenzhen Delica Medical Equipment Co., Ltd.) with a 1.6 MHz frequency setting was utilized. The pulsatility index of the middle cerebral artery was investigated via the temporal window.

### MRI procedures

Vital signs monitoring was performed before and immediately after DCE-MRI scans, including blood pressure and heart rates. Blood flow velocities and blood vessel diameters of the internal carotid arteries and the external carotid arteries were measured in NCs (*n* = 25), before-CPAP-treatment OSA (*n* = 11) and after-CPAP-treatment OSA (*n* = 13) groups using color Doppler ultrasound (Vivid E95, GE Healthcare).

All images were acquired using a 3 T MRI scanner (Skyra, Siemens Healthcare) with a standard 20-channel head coil for radiofrequency transmission.

The MRI protocol included the following sequences:

1. To visualize the PVSs and the ventricles, the 3D T2-weighted sequence was used with main sequence parameters as follows: TR/TE = 4200/110 ms, flip angle 150°, FOV 240 mm, acquisition matrix 320 × 270, 5.0 mm thickness and acquisition time of 46 s.
2. To detect the perfusion of the brain parenchyma from capillaries and the perivascular fluid flow dynamics patterns, and the following high-resolution MRI sequences were used.
  1. 3D T1-weighted sequence was used with main sequence parameters as follows: repetition time/echo time (TR/TE) = 190/2.6 ms, flip angle 70°, FOV 250 mm, acquisition matrix 288 × 230, 5.0 mm thickness and acquisition time of 44 s.
  2. T1 mapping sequence was used with main sequence parameters as follows: TR/TE = 5.3/2.4 ms, flip angle 2.99999984411°, FOV 240 mm, acquisition matrix 192 × 126, 5.0 mm thickness and acquisition time of 1 min 28 s.
  3. DCE-MRI: the 3D T1-vibe sequence was used with main sequence parameters as follows: TR/TE = 2.8/0.8 ms, flip angle 14.9999992205°, FOV 220 mm, acquisition matrix 128 × 128, 80 contiguous sections with 5 mm thickness and acquisition time of 9 min 59 s. For the DCE scan, the suggested dosage (0.1 mmol/kg^−1^) of gadobutrol (Gadovist, Bayer Pharma AG) was administered intravenously with an automatic high-pressure syringe (Spectris MRI Injector System, Medrad) for a stable injection speed.

### Motion correction

During head MRI scans, a series of measures were used to avoid movement artifacts, as previously described [22]:

1. Long-term averaging method was taken to reduce motion artifacts generated by swallowing and cerebral artery pulsation.
2. During MRI scans, the head of participants was fixed by placing folded towels in the head coil.
3. Behavioral interventions for reducing head motion during MRI scans. In detail, a cross logo was fixed in the middle and upper part of the machine and participants were told to stare at the fixation cross logo throughout the MRI scans. Those who could not complete the MRI scans or whose images were rated as blurred after motion correction were excluded from the analysis.

### MRI analysis

Three radiologists with a combined 10 years of expertise in MRI analysis analyzed all of the MRI data using commercial image viewing software (IntelliSpace Portal v.7, Philips Healthcare), post-processing software (syngoMMWP VE40A, Siemens AG), and Syngo.VIA. They were blinded to the patients’ information. Furthermore, data were analyzed independently by a trained team consisting of three board-certified neuroradiologists, who were kept in the dark regarding the patient's name and medical background.

Fiji/ImageJ software was used to analyze T2 axial pictures while obscuring patient data. Following binarization and inversion, these images were used to pinpoint the regions of interest (ROIs) of the PVSs in the frontal cortex and basal ganglia, the bilateral lateral ventricles, the fourth ventricle, the total brain parenchyma at the level of the frontal cortex, the total brain parenchyma of the bilateral basal ganglia, the total brain parenchyma at the level of the lateral ventricles, and the total cerebellum parenchyma at the level of the fourth ventricle. The relative area ratios of the PVSs in the bilateral frontal cortex and the basal ganglia were calculated from the area of PVSs in the frontal cortex or basal ganglia divided by the total brain area at the level of the frontal cortex or the total area of the bilateral basal ganglia. The relative area ratios of the lateral ventricles were calculated from the area of lateral ventricles divided by the total brain area at the level of the lateral ventricles. The relative area ratios of the fourth ventricle were calculated from the area of the fourth ventricle divided by the total cerebellum area at the level of the fourth ventricle, respectively.

Importing the data of DCE-MRI scan into TISSUE 4D software for motion correction and image registration, so that signal strength was converted to gadolinium concentration. DCE-MRI pseudo-color pictures were created automatically and combined with T2 images. The modified two-compartment Tofts model was fitted to the DCE-MRI images, and the arterial input function was chosen as medium. ROIs that outline the structure of PVSs were expertly and manually defined at the bilateral frontal cortex and basal ganglia according to T2-weighted MRI images by radiologists. The PVSs had high intensity as seen in T2-weighted images, while had low intensity in T1-weighted images [23]. Using concentration–time curves (CTCs), obtained DCE-MRI data of PVSs were derived and calculated characteristic parameters including peak concentration, wash-in rate, and wash-out rate. CTCs were generated by averaging contrast concentration within each ROI at different time points using the mean curve function in Syngo.VIA software. Each average CTC was derived from the average of the curves belonging to the corresponding groups using ggplot2 R package (https://ggplot2.tidyverse.org.). Clustering was performed using the K-means clustering algorithm (https://CRAN.R-project.org/package=factoextra) based on the feature parameters of CTCs including wash-in rate and peak concentrations.

### Statistical analysis

Statistical analyses were performed and Figures were illustrated using R (version 4.0.5), GraphPad Prism (version 8.0.0 for MacOS, GraphPad Software, La Jolla, CA, USA) and SPSS 26.0 (IBM). The clinical and demographic continuous data were represented by mean ± s.d. Demographic and clinical characteristics were compared using a chi-squared test and two-sided Mann–Whitney U-test. Normality testing and homogeneity tests of variance for all continuous variables were made before the analysis. The values of the relative area ratios of the bilateral lateral ventricles and the relative area ratios of the fourth ventricles in different groups were compared using a Mann–Whitney *U*-test, respectively. The values of the relative area ratios of the PVSs in the bilateral frontal cortex and the basal ganglia were compared using a Mann–Whitney *U*-test, respectively. The diagnostic accuracy was evaluated using ROC curve analysis. Spearman correlation was used to test the association among the relative area ratios of the bilateral lateral ventricles, the PVSs in the basal ganglia, the wash-out rate values of type I CTCs of frontal cortex of OSA patients, AHI and oxygen desaturation index (ODI) measured by PSG monitoring, and the MMSE scores, the MoCA scores, the PSQI scores and the ESS scores. (https://ggplot2.tidyverse.org.) (https://CRAN.R-project.org/package=ggridges.) DCE-MRI parameters in different groups were defined and visualized in clusters using K-means cluster analysis (https://CRAN.R-project.org/package=factoextra.) The best number of clusters was determined by using the elbow method [24] (http://www.jstatsoft.org/v61/i06/.) The values of DCE-MRI parameters from the CTCs in different groups were compared using a Mann–Whitney *U*-test, respectively. *P* < 0.05 was considered statistically significant.

## Results

### Demographics

**Fig. 1:**
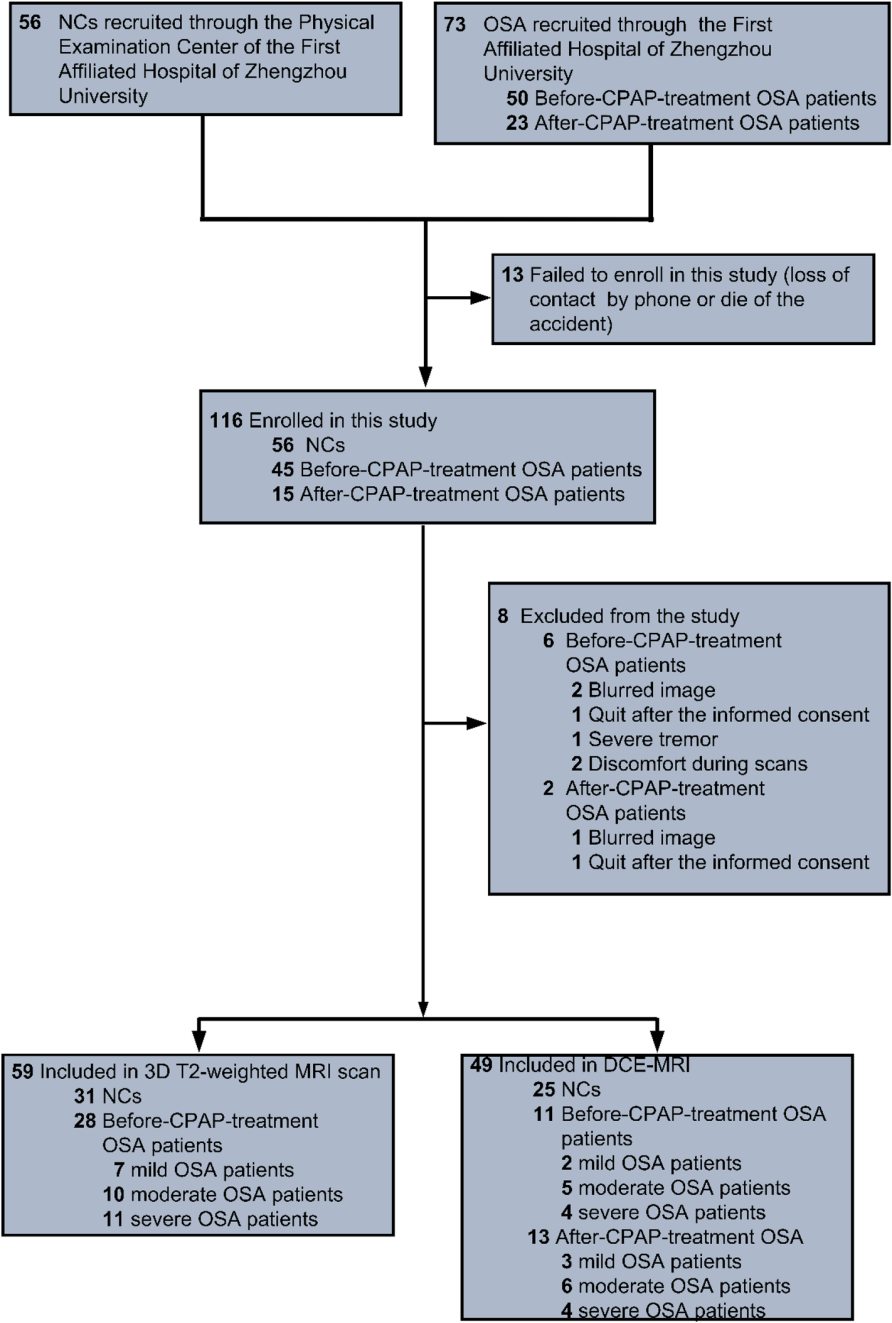
CONSORT diagram. Consolidated Standards of Reporting Trials flow diagram showing study participants screening, eligibility and inclusion

The demographic and clinical characteristics of the NCs and OSA are shown in the Consolidated Standards of Reporting Trials (CONSORT) flow diagram (Fig. 1, Table 1, Supplementary Table 1, Supplementary Table 2). 59 participants (NCs, *n* = 31; OSA, *n* = 28) completed 3D T2-weighted MRI scans to determine the relative area ratios of the perivascular spaces (PVSs) in the bilateral frontal cortex and the bilateral basal ganglia, and the relative area ratios of the lateral ventricles and the fourth ventricle. Dynamic contrast-enhanced MRI (DCE-MRI) scans were performed on 55 participants (NCs, *n* = 25; OSA before CPAP treatment, *n* = 11; OSA after CPAP treatment, *n* = 13).

**Table 1:** Demographics and clinical characteristics of the participants

	NCs	OSA
No. (% female)	56 (44.6%)	52 (42.3%)
Age, years	52.0 (11.7)	52.6 (10.0)
AHI	NA	28.9 (18.0)
ODI	NA	30.7 (20.8)
LSaO_2_ (%)	NA	79.7 (8.9)
BMI (kg/m^2^)	25.6 (2.0)	28.4 (2.4)
MMSE	NA	27.7 (2.3)
MoCA	NA	27.0 (2.4)
PSQI	NA	8.1 (3.7)
ESS	NA	6.3 (3.0)

Data are given as mean (SD). Mean (standard deviation) and N (%) were reported. Demographic factors and clinical characteristics were compared using chi-square test and two-sided Mann–Whitney tests

*OSA* obstructive sleep apnea, *NCs* normal controls, *AHI* Apnea–hypopnea index, *ODI* Oxygen desaturation index, *LSaO*_*2*_ oxygen saturation, *MMSE* Mini Mental State Examination, *MoCA* Montreal Cognitive Assessment, *BMI* Body Mass Index, *PSQI* Pittsburgh Sleep Quality Index and, *ESS* Epworth sleepiness scale, *NA* not applicable

Furthermore, there were notable differences between the NCs and OSA groups in terms of systolic blood pressure (SBP), diastolic blood pressure (DBP), respiratory rate (RR) and heart rate (HR) either before or after MRI scans (Supplementary Fig. 1). We assessed the diameters of the internal and external carotid arteries, as well as the pulse index of the middle cerebral arteries, to determine the participants' hemodynamic state. The hemodynamic parameters of the OSA group were not significantly different from those of the NCs group, according to the statistical analysis. (Supplementary Fig. 1).

### PVS and ventricular morphology changes and the correlation with cognitive impairment in OSA patients

**Fig. 2:**
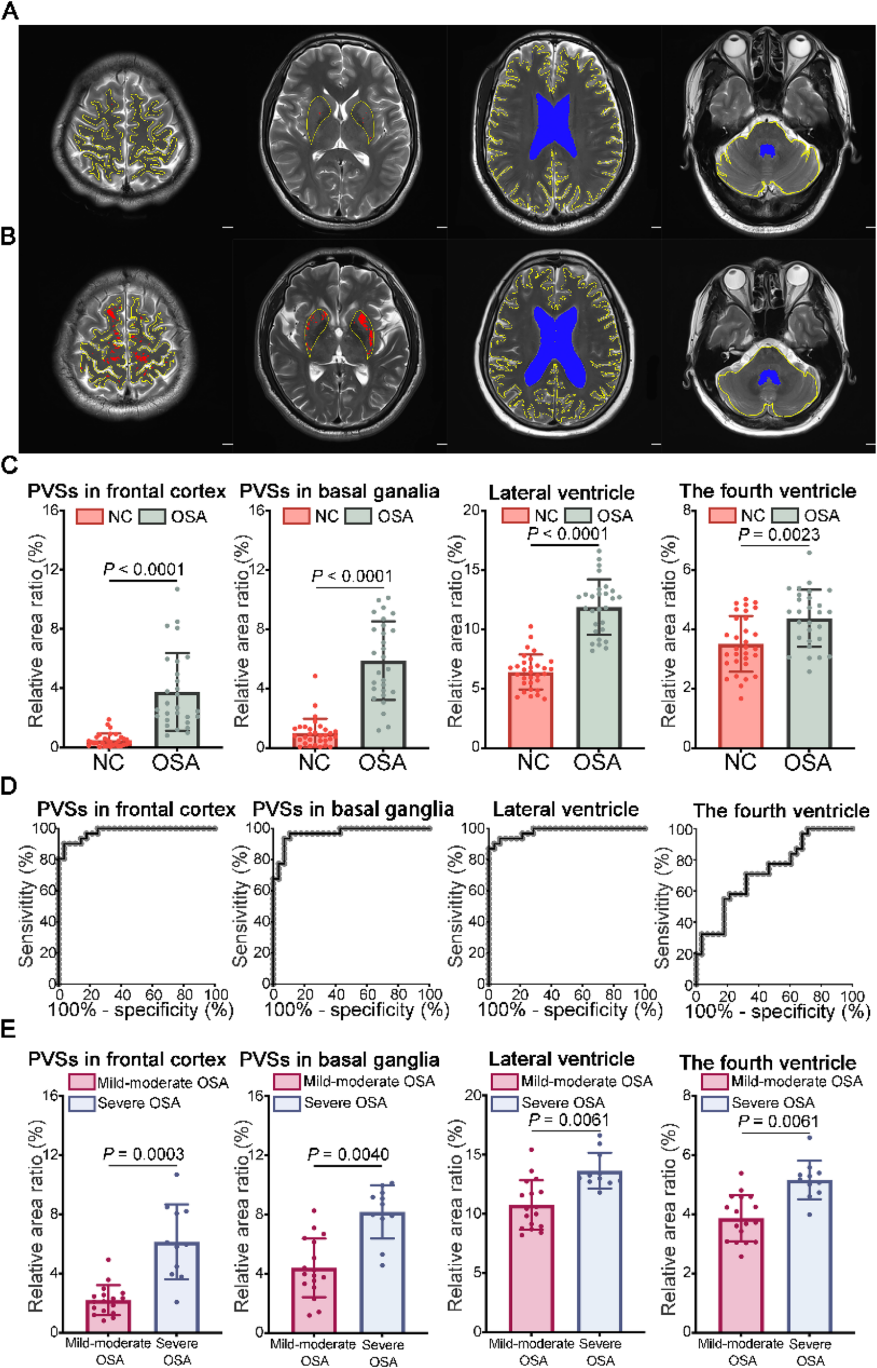

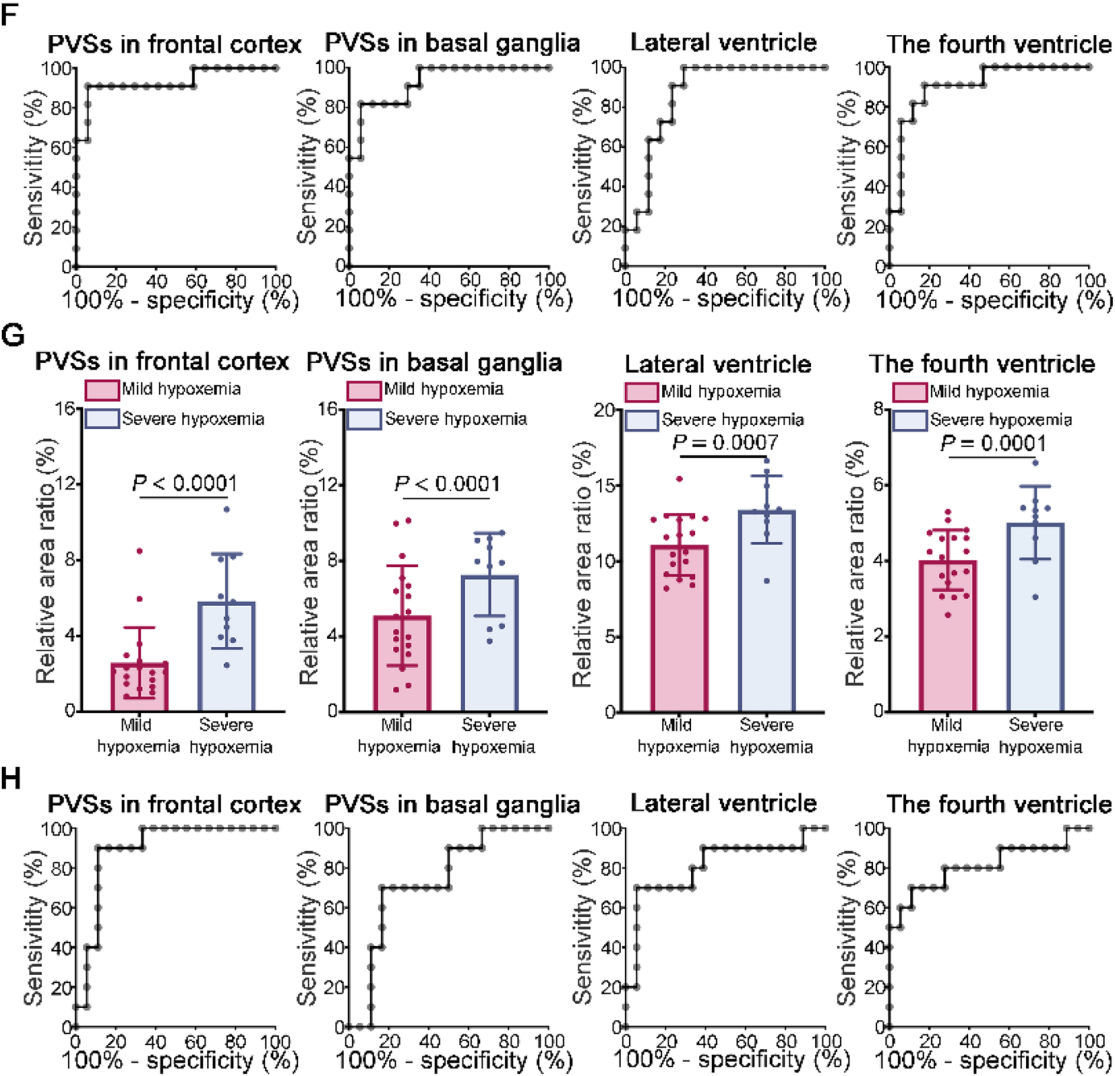
Measurement of the PVSs and ventricles areas by MRI. (**A**) Representative T2 axial MRI scans of PVSs in the bilateral frontal cortex and the bilateral basal ganglia of the NCs. The irregular regions within the closed yellow contour represent the frontal cortex or bilateral basal ganglia. The irregular red regions represent the PVSs. Representative T2 axial MRI scans of the bilateral lateral ventricle of the NCs. The irregular blue regions represent the bilateral lateral ventricles. The irregular regions within the closed yellow contour represent the total brain area at the level of the lateral ventricle. Representative images of the fourth ventricle of the NCs. The irregular blue regions represent the fourth ventricle. The irregular regions within the closed yellow contour represent the total cerebellum area at the level of the fourth ventricle. Scale bar, 20 mm. (**B**) Representative T2 axial MRI scans of PVSs in the bilateral frontal cortex and the bilateral basal ganglia, and the bilateral lateral ventricle and the fourth ventricle of the OSA. Scale bar, 20 mm. (**C**) Comparison of the relative area ratios of the bilateral frontal cortex, basal ganglia, lateral ventricle and the fourth ventricles between the NCs (*n* = 28) and OSA (*n* = 31) groups (Mann–Whitney *U*-test). (**D**) ROC curves of the relative area ratios of the bilateral frontal cortex, basal ganglia, lateral ventricle and the fourth ventricles for distinguishing OSA (*n* = 31) from NCs (*n* = 28). (**E**) Comparison of the relative area ratios of the bilateral frontal cortex, basal ganglia, lateral ventricle and the fourth ventricles between the mild-moderate OSA (*n* = 17) and severe OSA (*n* = 11) groups (Mann–Whitney *U*-test). (**F**) ROC curves of the relative area ratios of the bilateral frontal cortex, basal ganglia, lateral ventricle and the fourth ventricles for distinguishing severe OSA (*n* = 11) from mild-moderate OSA (*n* = 17). (**G**) Comparison of the relative area ratios of the bilateral frontal cortex, basal ganglia, lateral ventricle and the fourth ventricles between the OSA with mild hypoxemia (*n* = 18) and OSA with severe hypoxemia (*n* = 10) groups (Mann–Whitney *U*-test). (**H**) ROC curves of the relative area ratios of the bilateral frontal cortex, basal ganglia, lateral ventricle and the fourth ventricles for distinguishing OSA with severe hypoxemia (*n* = 10) from OSA with mild hypoxemia (*n* = 18)

Morphological changes of PVSs in the bilateral frontal cortex and basal ganglia were first assessed using the axial T1-weighted MR images in NCs and OSA groups (Fig. 2A,B). When comparing the OSA group to the NCs group, statistical analysis revealed that the relative area ratios of PVSs in both the bilateral frontal cortex and the basal ganglia were significantly higher in the OSA group (Fig. 2C). Then, the PVSs in the bilateral frontal cortex and basal ganglia between mild-moderate OSA and severe OSA groups were compared. The statistical results showed that the relative area ratios of PVSs in both the bilateral frontal cortex and the basal ganglia were significantly higher in the severe OSA group than in the mild-moderate OSA group (Fig. 2E). Furthermore, PVSs in the bilateral frontal cortex and basal ganglia were also compared between OSA with mild hypoxemia and OSA with severe hypoxemia groups. The OSA with severe hypoxemia group had higher relative area ratios of PVSs in both the bilateral frontal cortex and the basal ganglia compared to those in OSA with mild hypoxemia group (Fig. 2G). Next, receiver operating characteristic (ROC) curve analysis was used to evaluate the diagnostic accuracy of the PVSs in the bilateral frontal cortex and basal ganglia to discriminate between OSA and NCs. The results revealed that the relative area ratios of PVSs in the bilateral frontal cortex (sensitivity of 90.32%, specificity of 96.43%, AUROC of 0.9781, *P* < 0.0001) and the bilateral basal ganglia (sensitivity of 93.55%, specificity of 92.86%, AUROC of 0.9677, *P* < 0.0001) (Fig. 2D, Supplementary Table 3) all had the adequate accuracy to distinguish OSA from NCs (Fig. 2D, Supplementary Table 3). In addition, the relative area ratios of PVSs in the bilateral frontal cortex (sensitivity of 90.91%, specificity of 94.12%, AUROC of 0.9305, *P* = 0.0002) and in the bilateral basal ganglia (sensitivity of 81.82%, specificity of 94.12%, AUROC of 0.9251, *P* = 0.0002) all had the adequate accuracy to distinguish severe OSA from mild-moderate OSA (Fig. 2F, Supplementary Table 3). Moreover, the relative area ratios of PVSs in the bilateral frontal cortex (sensitivity of 90.00%, specificity of 88.89%, AUROC of 0.8944, *P* = 0.0007) and in the bilateral basal ganglia (sensitivity of 70.00%, specificity of 83.33%, AUROC of 0.7389, *P* = 0.0392) all had the appropriate accuracy to distinguish OSA with severe hypoxemia from OSA with mild hypoxemia (Fig. 2H, Supplementary Table 3).

To further evaluate the CSF circulation, the relative area ratios of both bilateral lateral ventricles and the fourth ventricle were measured using the axial T2 sequence (Fig. 2A, B). The findings showed that the relative area ratios of both the bilateral lateral ventricles and the fourth ventricle in the OSA group were increased compared with those in the NCs group (Fig. 2C). Additionally, the severe OSA group has higher relative area ratios of both the bilateral lateral ventricles and the fourth ventricle than mild-moderate OSA group (Fig. 2E). Besides, in the OSA with severe hypoxemia group, the relative area ratios of both the bilateral lateral ventricles and the fourth ventricle are higher than in the OSA with mild hypoxemia group (Fig. 2G). Then, ROC curve analysis showed that the relative area ratios of the bilateral lateral ventricles (sensitivity of 87.10%, specificity of 100.0%, AUROC of 0.9804, *P* < 0.0001) and the fourth ventricle (sensitivity of 70.97%, specificity of 67.86%, AUROC of 0.7281, *P* = 0.0027) all showed the desirable accuracy to distinguish OSA from NCs (Fig. 2D, Supplementary Table 3). In addition, the relative area ratios of the bilateral lateral ventricles (sensitivity of 100.0%, specificity of 70.59%, AUROC of 0.8663, *P* = 0.0013) and the fourth ventricle (sensitivity of 90.91%, specificity of 82.35%, AUROC of 0.9037, *P* = 0.004) all had the adequate accuracy to distinguish severe OSA from mild-moderate OSA (Fig. 2F, Supplementary Table 3). Moreover, the relative area ratios of the bilateral lateral ventricles (sensitivity of 70.00%, specificity of 94.44%, AUROC of 0.7170, *P* < 0.0001) and the fourth ventricle (sensitivity of 70.00%, specificity of 88.89%, AUROC of 0.8111, *P* = 0.0073) all showed appropriate accuracy for distinguishing OSA with severe hypoxemia from OSA with mild hypoxemia (Fig. 2H, Supplementary Table 3).

Next, Spearman correlation was used to test the correlations among morphological changes of PVSs and cerebral ventricle, the AHI and the ODI measured by PSG monitoring, the MMSE scores and the MoCA scores. The correlation analysis indicated that the relative area ratios of PVSs in both the bilateral frontal cortex and the basal ganglia were positively correlated with the relative area ratios of both bilateral lateral ventricles and the fourth ventricle, respectively (Fig. 4A). In addition, the AHI showed a strong positive correlation with the relative area ratios of PVSs in both the bilateral frontal cortex, and moderate positive correlations with the relative area ratios of PVSs in both the bilateral basal ganglia and the relative area ratios of both bilateral lateral ventricles and the fourth ventricle (Fig. 4A). Moreover, the ODI showed moderate positive correlations with the relative area ratios of PVSs in both the bilateral frontal cortex and the basal ganglia, and the relative area ratios of the fourth ventricle (Fig. 4A). Furthermore, the MMSE score was strongly negatively correlated with the relative area ratios of PVSs in both the bilateral frontal cortex, and moderately negatively correlated with the relative area ratios of PVSs in both the bilateral basal ganglia and the relative area ratios of both bilateral lateral ventricles and the fourth ventricle (Fig. 4A), while the MoCA score was strongly negatively correlated with the relative area ratios of PVSs in both the bilateral frontal cortex, moderately negatively correlated with the relative area ratios of both bilateral lateral ventricles, and weakly negatively correlated with the relative area ratios of PVSs in both the bilateral basal ganglia and the relative area ratios of the fourth ventricle (Fig. 4A). Moreover, the PSQI score showed a moderate positive correlation with the relative area ratios of PVSs in both the bilateral frontal cortex and the bilateral basal ganglia, while the ESS score was moderate positively correlated with the relative area ratios of PVSs in both the bilateral frontal cortex and the relative area ratios of the fourth ventricle, and weakly positively correlated with the relative area ratios of PVSs in both the bilateral basal ganglia (Fig. 4A). Furthermore, the spearman correlation was also used to test the correlations among other PSG score and morphological changes of PVSs and ventricles. The correlation analysis showed that the apnea index (AI) was positively correlated with the relative area ratios of PVSs in both the bilateral frontal cortex and the relative area ratios of both bilateral lateral ventricles, while the time spent with SaO2 < 90% (T90) was positively correlated with morphological changes of PVSs and the cerebral ventricle (Supplementary Fig. 2). These findings demonstrated that there were correlations among morphological changes of PVSs, ventricles, sleep habits, and cognitive impairment in OSA patients. Moreover, the MMSE score was strongly negatively correlated with the AHI and the ODI and the MoCA score was moderately negatively correlated with the AHI and the ODI, while the PSQI score was moderately positively correlated with the AHI and the ODI, and the ESS score was strongly positively correlated with the AHI and the ODI, which indicated that clinical manifestations were also correlated with the cognitive impairment in OSA patients.

### Impaired glymphatic drainage and correlation with intermittent attacks and cognitive impairment in OSA patients

**Fig. 3:**
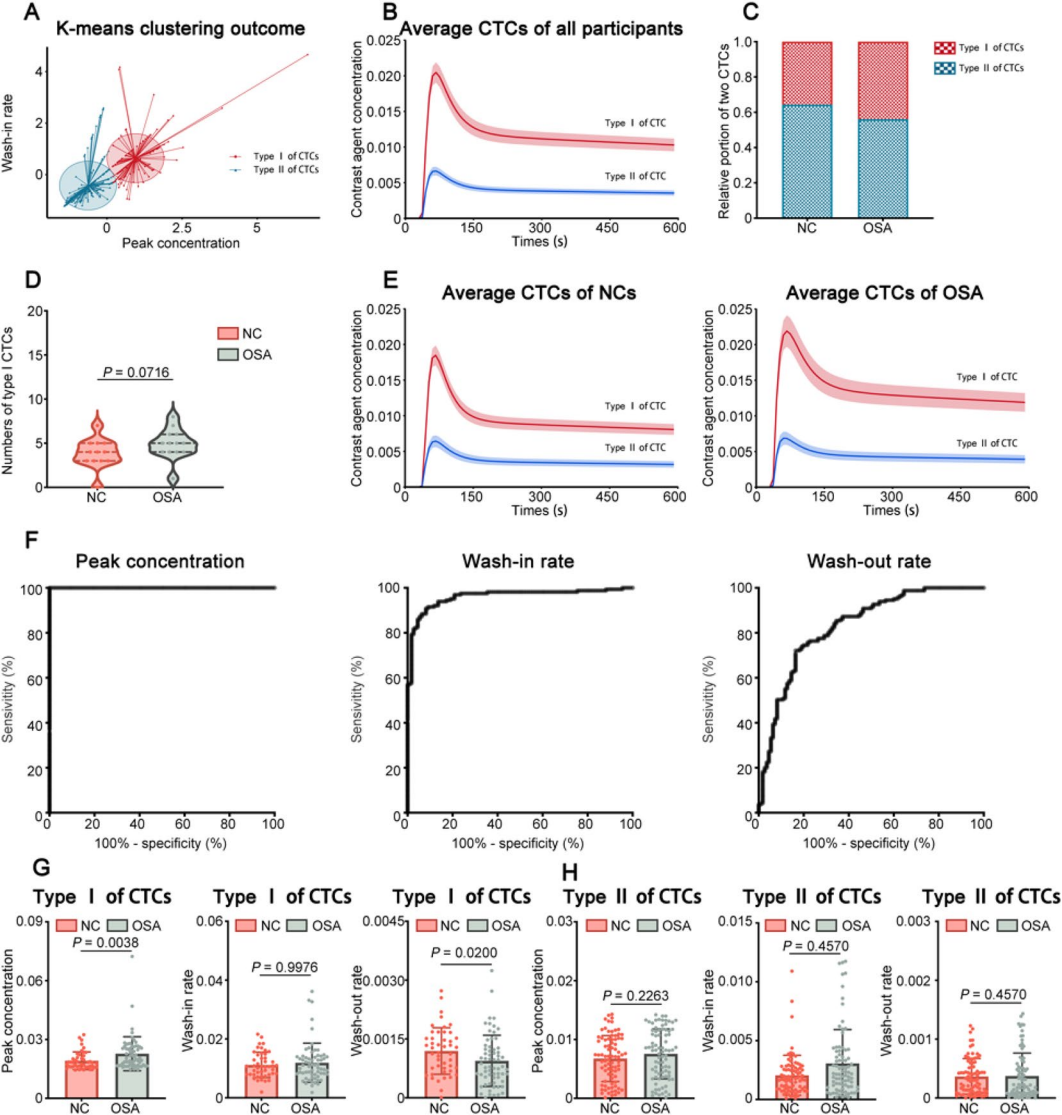

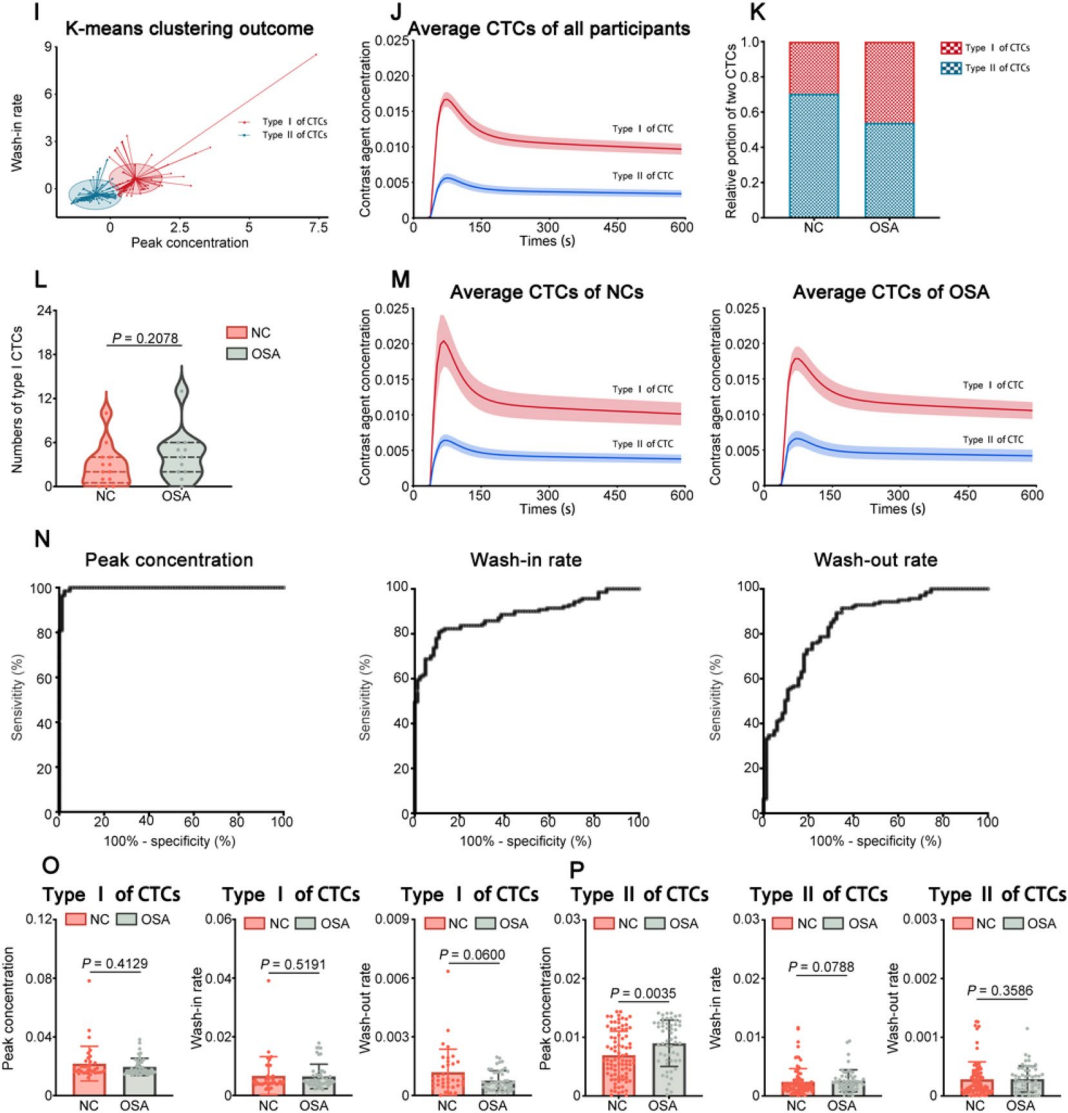
Measurement of fluid flow of PVSs by DCE-MRI. (**A**) K-means cluster outcome of all CTCs in the bilateral frontal cortex of the NCs (*n* = 236) and before-CPAP-treatment OSA (*n* = 253) groups. The red drops represent type I CTCs. The blue triangles represent type II CTCs. (**B**) Average CTCs based on the cluster analysis of totaling 489 CTCs from the NCs (*n* = 236) and before-CPAP-treatment OSA (*n* = 253) groups. (**C**) The proportions of the type I and type II CTCs in NCs (*n* = 236) and before-CPAP-treatment OSA (*n* = 253) groups. (**D**) The numbers of type I CTCs for each subject of NCs (*n* = 236) and before-CPAP-treatment OSA (*n* = 253) groups. (**E**) The average CTCs of the NCs and before-CPAP-treatment OSA groups. (**F**) ROC curves of peak concentration values wash-in rate values, and wash-out rate values of all CTCs in frontal cortex for distinguishing type II CTCs (*n* = 314) from type I CTCs (*n* = 175). (**G**) Comparison of peak concentration values wash-in rate values, and wash-out rate values of the type I CTCs in the frontal cortex between NCs (*n* = 70) and before-CPAP-treatment OSA (*n* = 105) groups (Mann–Whitney *U*-test). (**H**) Comparison of peak concentration values, wash-in rate values, and wash-out rate values of the type II CTCs in the frontal cortex between NCs (*n* = 166) and before-CPAP-treatment OSA (*n* = 148) groups (Mann–Whitney *U*-test). (**I**) K-means cluster outcome of all CTCs in the bilateral frontal cortex of the NCs (*n* = 236) and after-CPAP-treatment OSA (*n* = 253) groups. The red drops represent type I CTCs. The blue triangles represent type II CTCs. (**J**) Average CTCs based on the cluster analysis of totaling 489 CTCs from the NCs (*n* = 236) and after-CPAP-treatment OSA (*n* = 253) groups. (**K**) The proportions of the type I and type II CTCs in NCs (*n* = 236) and after-CPAP-treatment OSA (*n* = 253) groups. (**L**) The numbers of type I CTCs for each subject of NCs (*n* = 236) and after-CPAP-treatment OSA (*n* = 253) groups. (**M**) The average CTCs of the NCs and after-CPAP-treatment OSA groups. (**N**) ROC curves of peak concentration values wash-in rate values, and wash-out rate values of all CTCs in frontal cortex for distinguishing type II CTCs (*n* = 314) from type I CTCs (*n* = 175). (**O**) Comparison of peak concentration values wash-in rate values, and wash-out rate values of the type I CTCs in the frontal cortex between NCs (*n* = 70) and after-CPAP-treatment OSA (*n* = 105) groups (Mann–Whitney *U*-test). (**P**) Comparison of peak concentration values wash-in rate values, and wash-out rate values of the type II CTCs in the frontal cortex between NCs (*n* = 166) and after-CPAP-treatment OSA (*n* = 148) groups (Mann–Whitney *U*-test)

To detect the fluid dynamics in the glymphatic drainage system, DCE-MRI was utilized to identify the fluid flow of PVSs of bilateral frontal cortex, and CTCs were extracted by tracing the borders of all the visible PVSs. Two types of CTCs were recognized using k-means cluster analysis based on feature characteristics including wash-in rates and peak concentrations in the NCs and OSA groups. The type I CTCs showed a steeper climbing slope and greater peak concentration than the type II CTCs, which was compatible with the fluid flow patterns in the PASs. In contrast, the type II CTCs had a lower ascending slope and peak concentration, which depicted the fluid flow patterns in the PVESs. We first identified 275 CTCs of the PVSs of frontal cortex by k-means cluster in the two groups (Fig. 3A). The average curves generated by all the CTCs of type I (the top) and type II (the bottom) in the frontal cortex were shown in Fig. 3B.

The OSA group (43.84%) had a higher percentage of type I CTCs than the NCs group (35.66%) (Fig. 3C). In addition, the OSA group (each patient had an average of 4.923 CTCs) had considerably more type I CTCs than the NCs group (each patient had an average of 3.833 CTCs) (Fig. 3D). Furthermore, Fig. 3E depicted the average curves formed by all type I and type II CTCs in the frontal cortex in the NCs and OSA groups. The statistical results indicated that the peak concentration values of all CTCs in the frontal cortex had adequate accuracy for distinguishing type II CTCs from type I CTCs (sensitivity of 98.73%, specificity of 98.86%, AUROC of 0.9993, *P* < 0.0144) (Fig. 3F, Supplementary Table 4). In addition, the wash-in rate values of all CTCs in the frontal cortex showed desirable accuracy for distinguishing type II CTCs from type I CTCs (sensitivity of 81.21%, specificity of 84.57%, AUROC of 08,761, *P* < 0.0036) (Fig. 3F, Supplementary Table 4). The wash-out rate values of all CTCs in the frontal cortex had appropriate accuracy for distinguishing type II CTCs from type I CTCs (sensitivity of 76.11%, specificity of 80.00%, AUROC of 0.8417, *P* < 0.0004) (Fig. 3F, Supplementary Table 4). Moreover, the OSA group had considerably higher peak concentration and lower wash-out rate values of type I CTCs than the NCs group (Fig. 3G). Besides, there were no differences of the peak concentration, the wash-in rate, and the wash-out rate values of type II CTCs between the OSA group and the NCs group (Fig. 3H).

**Fig. 4:**
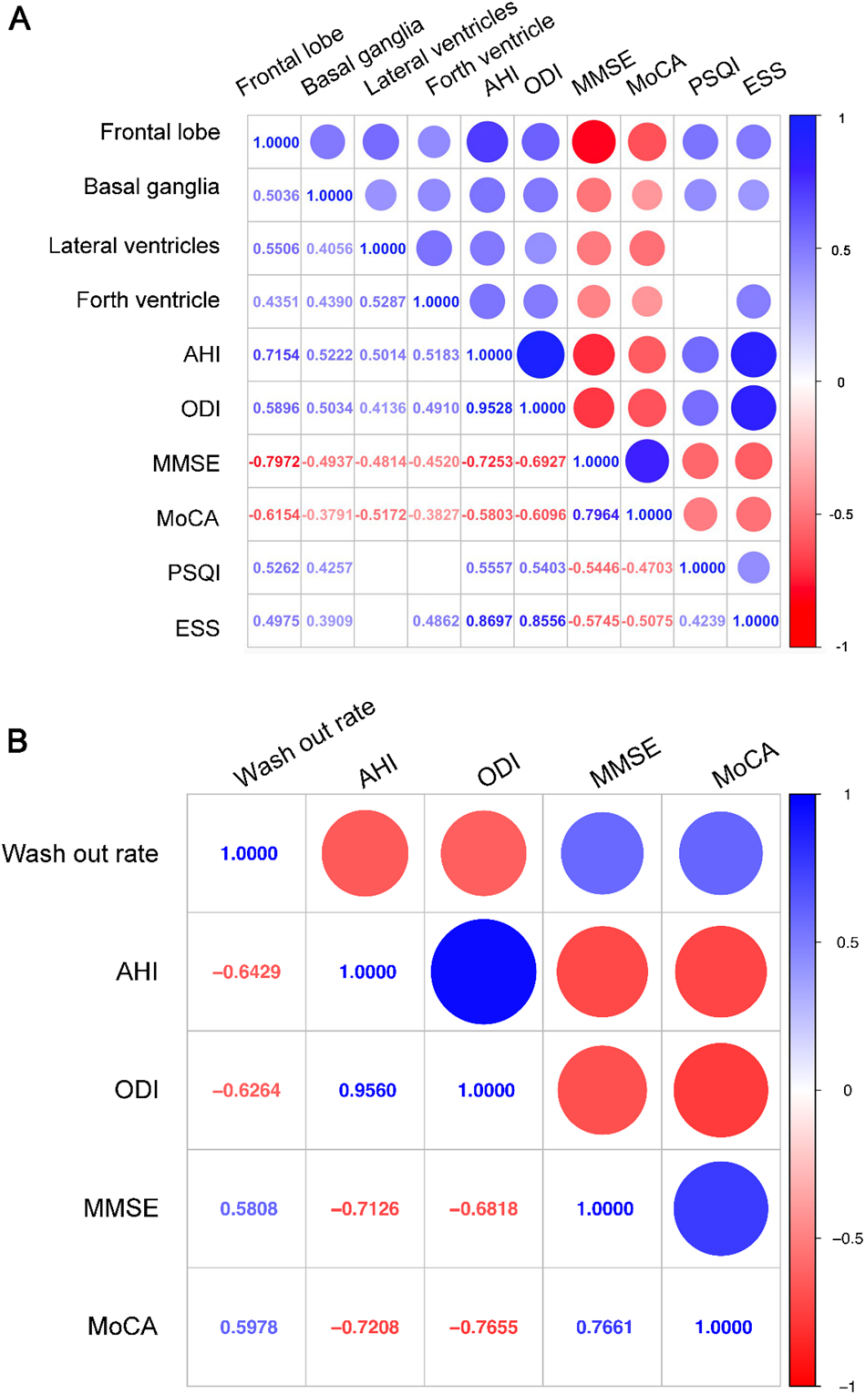
Spearman correlations between imaging parameters and clinical manifestation. (**A**) Heatmap of Spearman correlations among morphological changes of PVSs, ventricle enlargement, the AHI, the ODI, the MMSE scores, the MoCA scores, the PSQI scores and the ESS scores. The circle size and color intensity represent the magnitude of correlation. (**B**) Heatmap of Spearman correlations among wash-out rate values of type I CTCs of frontal cortex, the AHI, the ODI, the MMSE scores and the MoCA scores. The circle size and color intensity represent the magnitude of correlation

Next, correlation analysis was performed using Spearman correlation. The correlation analysis indicated that there were strongly negative correlations among the wash-out rate values of type I CTCs of frontal cortex, AHI and ODI in OSA patients (Fig. 4B). Moreover, the MMSE scores and the MoCA scores of OSA patients were moderately positively correlated with the wash-out rate values of type I CTCs, which demonstrated that the cognitive impairment in OSA patients was associated with the dysfunction of glymphatic drainage. (Fig. 4B).

### Improvement of glymphatic drainage in OSA patients after CPAP treatment

It has been indicated that treatment with CPAP could improve cognitive functions in OSA patients [17], however, the exact mechanism of this improvement is unknown. Using the k-means cluster, we identified total 184 CTCs in the PVSs of the frontal cortex in OSA patients and the NCs after 1 month CPAP treatment (Fig. 3I). The average curves generated by all the CTCs of type I and type II were shown in Fig. 3J. Type I CTCs were found at a larger proportion in the OSA group (46.08%) than in the NCs group (29.51%) after CPAP treatment (Fig. 3K). Furthermore, type I CTCs were significantly higher in OSA group (each patient had an average of 4.273 CTCs) than in NCs group (each patient had an average of 2.769 CTCs) after CPAP treatment (Fig. 3L). Additionally, Fig. 3M showed the average curves generated by all the CTCs of type I and type II in the two groups. According to the statistics, the peak concentration values of all CTCs in the basal ganglia showed adequate accuracy for distinguishing type II CTCs from type I CTCs (sensitivity of 95.67%, specificity of 98.68%, AUROC of 0.9969, *P* < 0.0321) (Fig. 3N, Supplementary Table 4). In addition, the wash-in rate values of all CTCs in the basal ganglia had desirable accuracy for distinguishing type II CTCs from type I CTCs (sensitivity of 76.17%, specificity of 90.79%, AUROC of 0.8889, *P* < 0.0086) (Fig. 3N, Supplementary Table 4). The wash-out rate values of all CTCs in the basal ganglia also had appropriate accuracy for distinguishing type II CTCs from type I CTCs (sensitivity of 81.23%, specificity of 70.39%, AUROC of 0.8408, *P* < 0.0020) (Fig. 3N, Supplementary Table 4). Moreover, no significant differences between the two groups were detected in any of the type I CTC parameters (peak concentration, wash-in rate or wash-out rate) (Fig. 3O). However, the OSA patients had considerably higher peak concentration values of type II CTCs than those of NCs group after CPAP treatment, while there were no significant differences in the wash-in rate or wash-out rate values between the OSA and NCs groups (Fig. 3P).

## Discussion

The glymphatic drainage system is a key waste clearance network made up of normally functioning PVSs, which provides a fluid channel for metabolites and neurotoxic waste products to be removed from the brain [1, 25, 26]. Previous studies put forward that glymphatic system dysfunction in OSA evidenced by DTI-ALPS [27, 28]. Unfortunately, there are no methods available to directly quantitatively assess the fluid dynamics of the glymphatic drainage system, including the inflow and outflow of brain interstitial fluid. In the present study, using DCE-MRI, the CTCs of PVSs were retrieved to quantify the perfusion and drainage of gadobutrol in PVSs, which exactly represented the glymphatic drainage function. Gadobutrol from arterioles would drain via PASs to the brain parenchyma, whereas gadobutrol from venules would drain through PVESs to the subarachnoid space. The wash-in rate and peak concentration values were utilized as CTC characteristic parameters, which demonstrated the physiological differences between PASs and PVESs. Moreover, the wash-out rate values of the PASs represented the drainage from the PASs to brain tissue. Besides, the type II CTCs ware detected in both NCs and OSA groups, which suggested that the PVESs could be found in normal aged individuals [29]. Our findings indicated that the glymphatic drainage system was markedly impaired in OSA patients. Furthermore, we tried to identify the improvement of glymphatic drainage function in OSA patients after 1 month CPAP treatment. We considered that the higher peak concentration value of OSA patients before CPAP treatment may due to the hypertension of the patients, and the slower wash-out rate value of OSA patients before CPAP treatment proved the more serious impairment of glymphatic drainage. After CPAP treatment, there were no differences between the OSA group and the NCs group in the peak concentration value and the wash-out rate value. Unfortunately, one limitation of these methods is that how to consider the CPAP treatment as adequate to improve the glymphatic drainage system. However, these limitations notwithstanding, this study does suggest that the CPAP treatment could improve the fluid dynamics of the glymphatic drainage system and thus improve the clinical symptoms and the cognitive function in OSA patients. Additionally, we found that morphological changes of PVSs occurred in OSA group as compared to the NCs group. What’s more, our data uncovered that the enlarged PVSs could also be found more significantly in OSA patients with severe hypoxemia than mild hypoxemia. In conjunction with the previous studies that the glial metabolism and the vascular unit of the glymphatic drainage system are altered in hypoxic conditions [30], we speculate that the hypoxic condition during sleep may also cause enlarged PVSs and glymphatic drainage system dysfunction in patients of OSA.

We further explored the potential relationship between the glymphatic drainage dysfunction and the cognitive decline in OSA patients. The results suggested the potential causal association of the outflow dysfunction of glymphatic drainage system and the cognitive decline. Previous research has shown that sleep fragmentation of OSA impede sleep-associated glymphatic circulation of CSF-ISF exchange, which may lead to hydrocephalus [31]. Ventricle enlargement was detected in the OSA group in this study, which was more noticeable in severe OSA or OSA with severe hypoxemia. These findings further proved that glymphatic drainage system dysfunction occurred in patients of OSA, especially in patients with severe OSA or hypoxemia. It was previously reported that dilated PVSs is linked to an increased risk of dementia [5, 32]. Our results showed that the MMSE score and the MoCA score were negatively associated with the enlarged PVSs, the expanded ventricles, and the AHI and the ODI scores, which further suggested that patients with OSA have a higher risk of cognitive impairment due to the glymphatic drainage system dysfunction.

## Conclusion

We found that glymphatic drainage system dysfunction occurred in OSA patients, including enlarged PVSs, ventricle expansion, and abnormal fluid dynamics of PVSs, which might be important imaging markers. In addition, the correlation between glymphatic drainage system dysfunction and hypoxia severity or cognitive decline, suggesting that impaired glymphatic drainage might contribute to cognitive decline via OSA-induced chronic intermittent hypoxia in OSA patients. Moreover, the fluid dynamics of the glymphatic drainage system was improved by CPAP treatment, indicating the novel therapeutic mechanism of CPAP treatment for cognitive function in OSA patients.

## Supplementary Information

Below is the link to the electronic supplementary material.

Supplementary file1 (DOCX 788 KB)

## Data Availability

All relevant data supporting the findings of this study are either included within the article and Supplementary Materials or are available upon request from the corresponding author.
