# Supplementary material for: Impaired glymphatic drainage underlying obstructive sleep apnea is associated with cognitive dysfunction
Source: J Neurol. 2023 Jan 20;270(4):2204–16. doi: 10.1007/s00415-022-11530-z (PMC10025229; doi:10.1007/s00415-022-11530-z)
Supplement: Supplementary file 1 — Supplementary file1 (DOCX 788 KB) [file 415_2022_11530_MOESM1_ESM.docx]

**Supplementary Materials**

**Supplementary Tables**

**Supplementary Table 1 Demographics and Clinical characteristics of the participants with MRI assessment**

|  | **NCs** | **Before-CPAP-treatment OSA** |
| --- | --- | --- |
| No. (% female) | 31 (45.2%) | 28 (46.4%) |
| Age, years | 50.8 (12.0) | 52.3 (10.1) |
| AHI | NA | 30.2 (18.8) |
| ODI | NA | 32.3 (21.4) |
| LSaO_2_ (%) | NA | 79.9 (9.5) |
| BMI (kg/m^2^) | 25.9 (2.1) | 28.8 (2.3) |
| MMSE | NA | 27.8 (2.3) |
| MoCA | NA | 27.0 (2.3) |
| PSQI | NA | 8.4 (4.0) |
| ESS | NA | 6.3 (3.0) |

Abbreviation: OSA, Obstructive sleep apnea; NCs, normal controls; AHI, Apnea–hypopnea index; ODI, Oxygen desaturation index; LSaO_2_, oxygen saturation; MMSE, Mini Mental State Examination; MoCA, Montreal Cognitive Assessment; BMI, Body Mass Index; PSQI, Pittsburgh Sleep Quality Indexand; ESS, Epworth sleepiness scale; NA, not applicable.

Data are given as mean (SD). Mean (standard deviation) and N (%) were reported. Demographic factors and clinical characteristics were compared using chi-square test and two-sided Mann-Whitney tests.

**Supplementary Table 2 Demographics and Clinical characteristics of the participants with DCE-MRI**

|  | **NCs** | **Before-CPAP-treatment OSA** | **After-CPAP-treatment OSA** |
| --- | --- | --- | --- |
| No. (% female) | 25 (44.0%) | 11 (45.5%) | 13 (46.2%) |
| Age, years | 53.4 (11.2) | 52.5 (10.2) | 53.5 (9.6) |
| AHI | NA | 27.9 (17.6) | 27.1 (16.5) |
| ODI | NA | 28.3 (20.3) | 29.1 (19.5) |
| LSaO_2_ (%) | NA | 79.9 (8.0) | 79.2 (8.1) |
| BMI (kg/m^2^) | 25.3 (1.9) | 27.6 (3.3) | 28.3 (1.5) |
| MMSE | NA | 27.7 (2.6) | 27.3 (2.3) |
| MoCA | NA | 26.9 (2.2) | 26.6 (2.5) |
| PSQI | NA | 8.9 (3.0) | 7.0 (3.2) |
| ESS | NA | 6.6 (2.5) | 5.8 (3.1) |

Abbreviation: OSA, Obstructive sleep apnea; NCs, normal controls; AHI, Apnea–hypopnea index; ODI, Oxygen desaturation index; LSaO_2_, oxygen saturation; MMSE, Mini Mental State Examination; MoCA, Montreal Cognitive Assessment; BMI, Body Mass Index; PSQI, Pittsburgh Sleep Quality Indexand; ESS, Epworth sleepiness scale; NA,not applicable.

Data are given as mean (SD). Mean (standard deviation) and N (%) were reported. Demographic factors and clinical characteristics were compared using chi-square test and two-sided Mann-Whitney tests.

**Supplementary Table 3 Diagnostic accuracy of ventricle area% and PVS area%**

|  | **AUROC** | **95% CI (%)** | **Threshold** | **Sensitivity (%)** | **95% CI (%)** | **Specificity (%)** | **95% CI (%)** |
| --- | --- | --- | --- | --- | --- | --- | --- |
| **NCs vs. OSA** | | | | | | | |
| PVS area% in frontal cortex | 0.9781 | 0.9503 – 1.0000 | < 0.0001 | 90.32 | 75.10 – 96.65 | 96.43 | 82.29 – 99.82 |
| PVS area% in basal ganglia | 0.9677 | 0.9285 – 1.0000 | < 0.0001 | 93.55 | 79.28 – 98.85 | 92.86 | 77.35 – 98.73 |
| The lateral ventricle area% | 0.9804 | 0.9536 – 1.0000 | < 0.0001 | 87.1 | 71.15 – 94.87 | 100.00 | 87.94 – 100.00 |
| The fourth ventricle area% | 0.7281 | 0.6005 – 0.8558 | 0.0027 | 70.97 | 53.41 – 83.90 | 67.86 | 49.34 – 82.07 |
| **Mild-moderate OSA vs. Severe OSA** | | | | | | | |
| PVS area% in frontal cortex | 0.9305 | 0.8214 – 1.0000 | 0.0002 | 90.91 | 62.26 – 99.53 | 94.12 | 73.02 – 99.70 |
| PVS area% in basal ganglia | 0.9251 | 0.8290 – 1.0000 | 0.0002 | 81.82 | 52.30 – 96.77 | 94.12 | 73.02 – 99.70 |
| The lateral ventricle area% | 0.8663 | 0.7303 – 1.0000 | 0.0013 | 100.00 | 74.12 – 100.00 | 70.59 | 46.87 – 86.72 |
| The fourth ventricle area% | 0.9037 | 0.7875 – 1.0000 | 0.0004 | 90.91 | 62.26 – 99.53 | 82.35 | 58.97 – 93.81 |
| **OSA with mild hypoxemia vs. OSA with severe hypoxemia** | | | | | | | |
| PVS area% in frontal cortex | 0.8944 | 0.7705 – 1.0000 | 0.0007 | 90.00 | 59.58 – 99.49 | 88.89 | 67.20 – 98.03 |
| PVS area% in basal ganglia | 0.7389 | 0.5475 – 0.9303 | 0.0392 | 70.00 | 39.68 – 89.22 | 83.33 | 60.78 – 94.16 |
| The lateral ventricle area% | 0.8111 | 0.6234 – 0.9988 | 0.0073 | 70.00 | 39.68 – 89.22 | 94.44 | 74.24 – 99.72 |
| The fourth ventricle area% | 0.8111 | 0.6195 – 1.0000 | 0.0073 | 70.00 | 39.68 – 89.22 | 88.89 | 67.20 – 98.03 |

Abbreviation: OSA, Obstructive sleep apnea; NCs, normal controls; PVSs, perivascular space; AUROC, area under the receiver operating characteristic curves; CI, confidence interval.

The sensitivity, specificity, threshold, AUROC and 95% CI of the AUROC, sensitivity and specificity were calculated by ROC curve.

**Supplementary Table 4 Diagnostic accuracy of peak concentration, wash-in rate and wash-out rate for distinguishing type Ⅰ from type Ⅱ CTCs.**

|  | **AUROC** | **95% CI (%)** | **Threshold** | **Sensitivity (%)** | **95% CI (%)** | **Specificity (%)** | **95% CI (%)** |
| --- | --- | --- | --- | --- | --- | --- | --- |
| **NCs vs. Before-CPAP-treatment OSA** | | | | | | | |
| Peak concentration | 1.0000 | 1.0000 – 1.0000 | < 0.0001 | 100.00 | 97.72 – 100.00 | 100.00 | 96.63 – 100.00 |
| Wash-in rate | 0.9610 | 0.9385 – 0.9835 | < 0.0001 | 90.91 | 85.54 – 94.41 | 91.82 | 85.18 – 95.64 |
| Wash-out rate | 0.8310 | 0.7810 – 0.8810 | < 0.0001 | 72.12 | 64.84 – 78.40 | 83.64 | 75.61 – 89.39 |
| **NCs vs. After-CPAP-treatment OSA** | | | | | | | |
| Peak concentration | 0.9969 | 0.9921 – 1.0000 | < 0.0001 | 98.58 | 94.98 – 99.75 | 97.59 | 91.63 – 99.57 |
| Wash-in rate | 0.8816 | 0.8379 – 0.9252 | < 0.0001 | 80.85 | 73.57 – 86.49 | 89.16 | 80.66 – 94.19 |
| Wash-out rate | 0.8431 | 0.7895 – 0.8967 | < 0.0001 | 89.36 | 83.19 – 93.45 | 67.47 | 56.81 – 76.58 |

Abbreviation: OSA, Obstructive sleep apnea; NCs, normal controls; CTCs, concentration-time curves; AUROC, area under the receiver operating characteristic curves; CI, confidence interval.

The sensitivity, specificity, threshold, AUROC and 95% CI of the AUROC, sensitivity and specificity were calculated by ROC curve.

**Supplementary Figure**

**
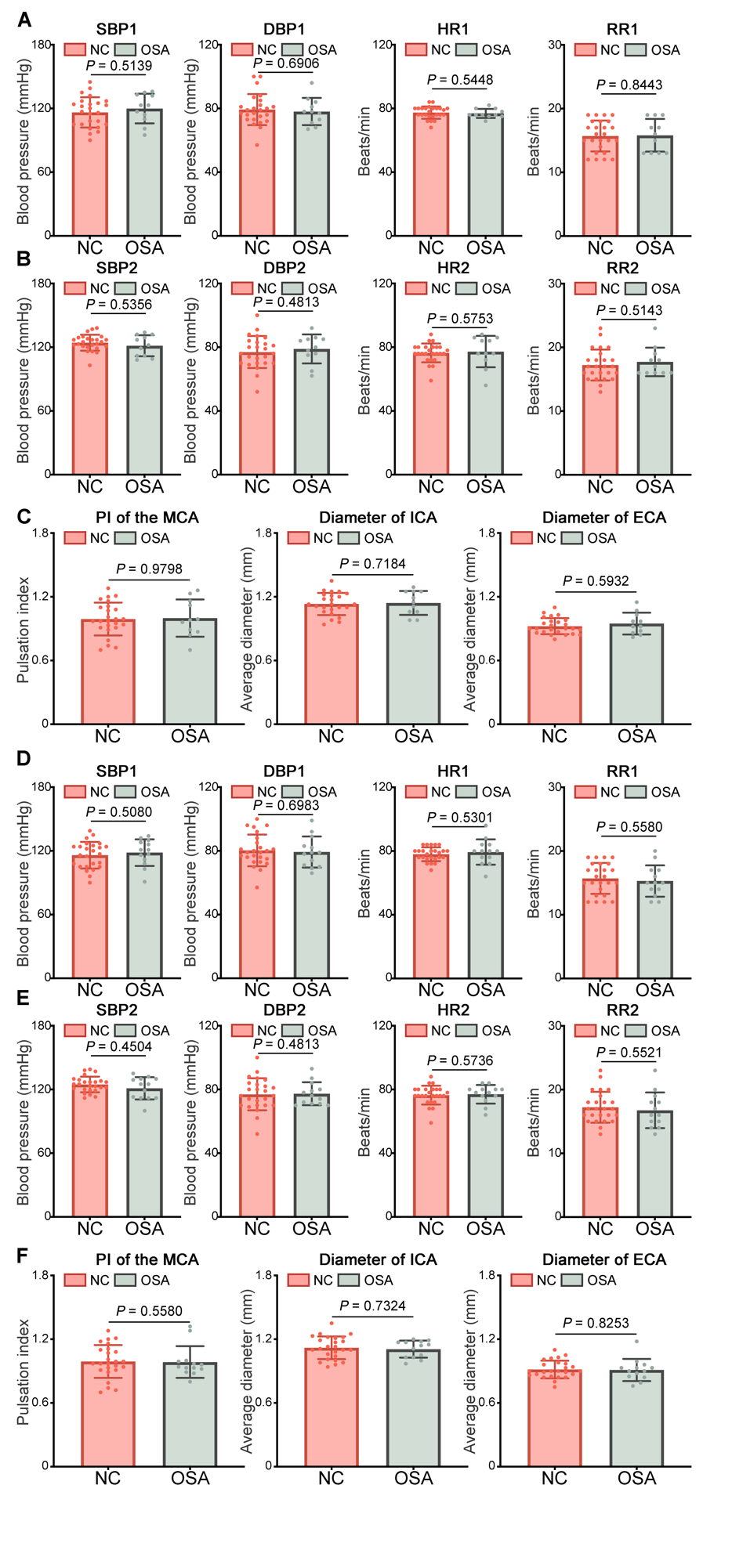
**

**Supplementary Fig.1 Statistical analysis of BP, HR, RR, pulsatility index, and blood vessel diameter in participants**

**(A)** Comparison of SBP, DBP, HR and RR before DCE-MRI scans between NCs (n = 25) and before-CPAP-treatment OSA (n = 11) groups (Mann-Whitney U-test). **(B)** Comparison of SBP, DBP, HR and RR after DCE-MRI scans between NCs (n = 25) and before-CPAP-treatment OSA (n = 11) groups (Mann-Whitney U-test). **(C)** Comparison of pulsatility index of MCA, blood vessel diameters of ECAs and ICAs between NCs (n = 25) and before-CPAP-treatment OSA (n = 11) groups (Mann-Whitney U-test). **(D)** Comparison of SBP, DBP, HR and RR before DCE-MRI scans between NCs (n = 25) and after-CPAP-treatment OSA (n = 13) groups (Mann-Whitney U-test). **(E)** Comparison of SBP, DBP, HR and RR after DCE-MRI scans between NCs (n = 25) and after-CPAP-treatment OSA (n = 13) groups (Mann-Whitney U-test). **(F)** Comparison of pulsatility index of MCA, blood vessel diameters of ECAs and ICAs between NCs (n = 25) and after-CPAP-treatment OSA (n = 13) groups (Mann-Whitney U-test).


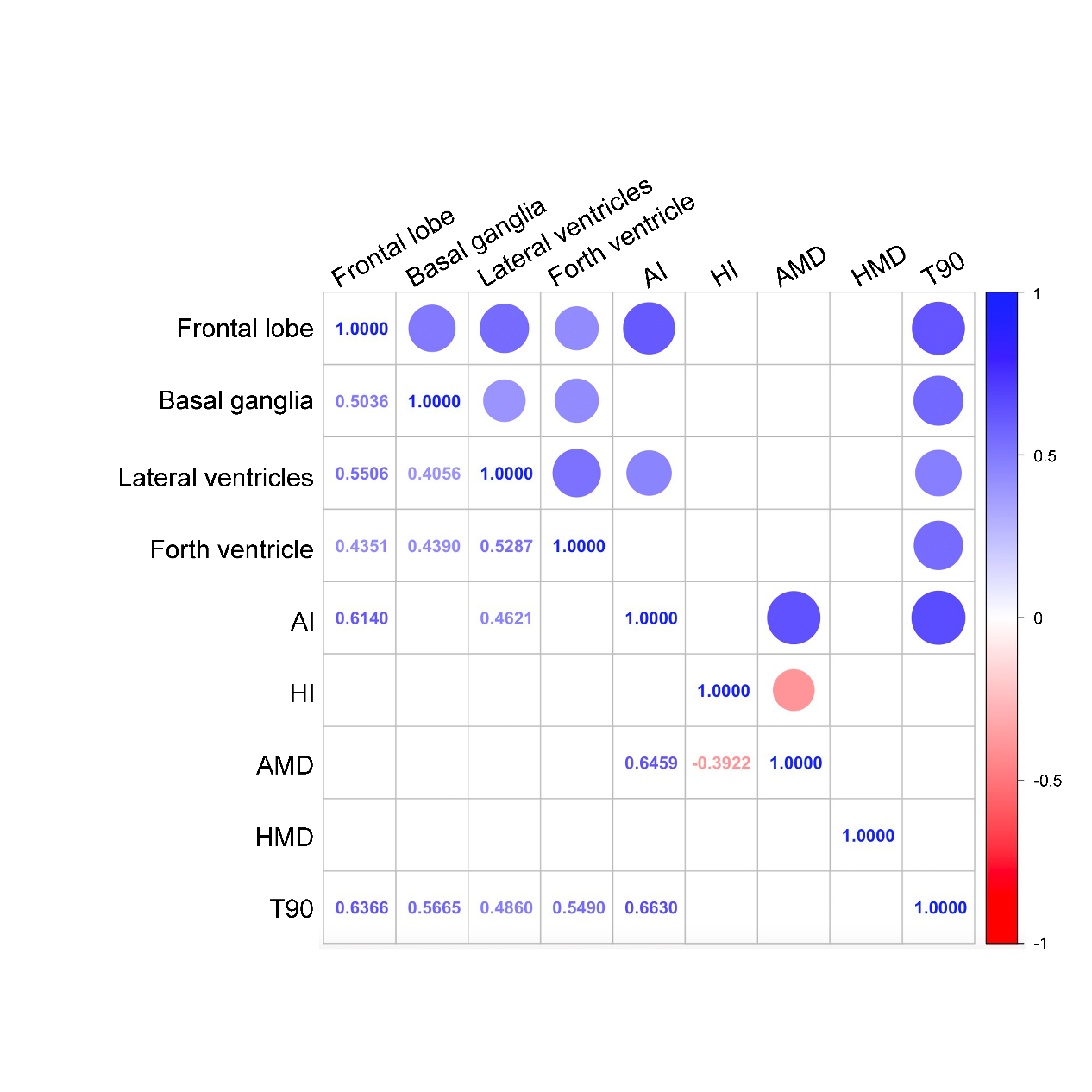


**Supplementary Fig.2 Spearman correlations between Imaging parameters and PSG scores**

Heatmap of Spearman correlations among morphological changes of PVSs, ventricle enlargement, the AI, the hypopnea index (HI), the apnea maximum duration (AMD), the hypopnea maximum duration (HMD) and the T90. The circle size and color intensity represent the magnitude of correlation
